# Novel Natural Structure Corrector of ApoE4 for Checking Alzheimer's Disease: Benefits from High Throughput Screening and Molecular Dynamics Simulations

**DOI:** 10.1155/2013/620793

**Published:** 2013-11-13

**Authors:** Manisha Goyal, Sonam Grover, Jaspreet Kaur Dhanjal, Sukriti Goyal, Chetna Tyagi, Sajeev Chacko, Abhinav Grover

**Affiliations:** ^1^Apaji Institute of Mathematics & Applied Computer Technology, Banasthali University, Tonk, Rajasthan 304022, India; ^2^School of Biotechnology, Jawaharlal Nehru University, New Delhi 110067, India; ^3^Department of Biotechnology, Delhi Technological University, New Delhi 110042, India; ^4^Thematic Unit of Excellence on Computational Materials Science, S. N. Bose National Centre for Basic Sciences, Sector III, Block JD, Salt Lake, Kolkata 700098, India

## Abstract

A major genetic suspect for Alzheimer's disease is the pathological conformation assumed by apolipoprotein E4 (ApoE4) through intramolecular interaction. In the present study, a large library of natural compounds was screened against ApoE4 to identify novel therapeutic molecules that can prevent ApoE4 from being converted to its pathological conformation. We report two such natural compounds PHC and IAH that bound to the active site of ApoE4 during the docking process. The binding analysis suggested that they have a strong mechanistic ability to correct the pathological structural orientation of ApoE4 by preventing repulsion between Arg 61 and Arg 112, thus inhibiting the formation of a salt bridge between Arg 61 and Glu 255. However, when the molecular dynamics simulations were carried out, structural changes in the PHC-bound complex forced PHC to move out of the cavity thus destabilizing the complex. However, IAH was structurally stable inside the binding pocket throughout the simulations trajectory. Our simulations results indicate that the initial receptor-ligand interaction observed after docking could be limited due to the receptor rigid docking algorithm and that the conformations and interactions observed after simulation runs are more energetically favored and should be better representations of derivative poses in the receptor.

## 1. Introduction

Alzheimer's disease (AD) is the most common form of dementia. AD is a harmful neurological disorder that affects about 5.4 million Americans of all ages [1]. One in every eight old Americans has AD, making it the sixth major cause of death in the United States [1]. In India, the annual incidence rate per 1,000 persons for AD is 11.67 for those above 55 years of age and even higher for those above 65 years [2]. AD, which affects memory, thinking ability, and behavior, is characterized by complex neuropathological features that include heaping of amyloid *β* (A*β*) followed by synaptic dysfunction, formation of neurofibrillary tangles, and elements of degenerating neurons [3]. Degeneration causes a decrease in the acetylcholine levels and in the activities of choline acetyltransferase [4]. 

Although the U.S. Food and Drug Administration (FDA) has approved 5 drugs that temporarily improve the condition of patients suffering from AD, none is fully effective because of associated toxic effects [1]. Tacrine, donepezil, rivastigmine, and memantine, for example, have significant side effects such as elevation of serum aminotransferase concentration, nausea, vomiting, diarrhea, anorexia, anxiety, and agitation [5–7]. The toxic effects of these drugs necessitate the development of new therapeutic compounds.

To develop a new drug, a computational approach is worthwhile and saves time. This approach involves screening new ligands for a specific target within a relatively short span of time. High throughput virtual screening (HTVS) is one of the most effective and rapid approaches for identifying probable inhibitors of the target protein [8]. Various potential drug targets have been reported to improve AD-associated pathological features such as acetylcholine esterases [9], NMDA receptor [10], and apolipoprotein E4 (ApoE4). ApoE plays a significant role in maintaining and repairing neurons. ApoE has three isoforms, namely, ApoE2, ApoE3, and ApoE4. The isoforms differ at residue positions 112 and 158 [11]. ApoE4 is the major genetic risk attributed to AD [12–17]. It acquires a pathological conformation through an intra-molecular interaction, in which positively charged Arg 112 repels the side chain of Arg 61 in the aminoterminal domain, allowing the formation of a salt bridge between Arg 61 and Glu 255 at the carboxyl terminal domain [18, 19]. Forty to eighty percent of patients with AD are estimated to possess at least one ApoE4 allele [20]. ApoE4 is less effective in maintaining and repairing neuronal cells compared to ApoE2 and ApoE3 [21–23]. ApoE4 also disrupts the normal process by which cells release excess A*β*, resulting in elevated levels of A*β* leading to its deposition in the brain [24–26]. ApoE4 uniquely performs neuron-specific proteolysis due to which harmful bioactive fragments are formed that can enter the cytosol, disrupt the mitochondrial energy balance, alter the cytoskeleton, and cause cell death [27–29]. ApoE is the only example of a susceptibility gene for AD [30] associated with lower glucose use and is believed to affect the hippocampus and cortex, areas found to be affected in patients with AD [31, 32]. It has been confirmed that the ApoE locus on chromosome 19 is strongly associated with the development of AD [12, 33, 34]. Small molecule structure correctors of ApoE4 have been suggested that effectively modulate the biophysical properties and the function of abnormal proteins. Some examples of ApoE4 structure correctors are GIND25 [35] and phthalazinone derivatives [36]. The evidential association of ApoE4 with increased risk of AD makes it a potential drug target for designing natural drug candidates for AD. 

The present study focuses on identifying potential natural drug candidates as structure correctors for ApoE4. Keeping this goal in mind, a large database of natural compounds was screened against the 3D structure of ApoE4 using high throughput technology. *In silico* screening led to the identification of a new class of ApoE4 structure correctors that abolish the ApoE4 domain interaction. The molecular dynamics (MD) were then simulated to examine the dynamic behavior of molecular interactions between the screened compounds and the functional residues of ApoE4. This study paves the way for the development of novel leads for AD treatment that have improved binding properties and pose low toxicity to humans.

## 2. Materials and Methods

### 2.1. Protein Preparation

The crystal structure of human ApoE4 [PDB ID: 1GS9], determined at a resolution of 1.70 Å, was retrieved from the Protein Data Bank [37]. ApoE4 contains a single domain of 22 kD. To preprocess the retrieved structure of ApoE4, Protein Preparation Wizard in Schrodinger's Maestro interface [38] was used, followed by optimization [39]. 

### 2.2. Grid Generation and Ligand Library Preparation

The prepared protein structure was used to generate a grid using the receptor grid generation utility of the Glide docking module of the Schrodinger suite [40, 41]. Residues Arg-61, Glu-109, and Arg-112 form the catalytic triad in the active cleft of ApoE4 [36, 42]. The ligand library was prepared by extracting approximately 0.2 million natural compounds from the ZINC database [43] and processing them with Schrodinger's LigPrep Wizard [44] and using the Lipinski filter. 

### 2.3. High Throughput Virtual Screening and Docking Studies

The prepared ligand library was screened with the Glide Program [41, 45]. Glide uses a systematic method for virtual screening based on incremental construction searching and provides the output as the GScore scoring function combined with various other parameters. Glide's HTVS and extraprecision (XP) algorithms combine to perform docking [46]. The screening against ApoE4 at the desired grid coordinates was performed through the HTVS docking algorithm [40]. Compounds with a significant docking score were subjected to Glide XP, a more precise docking algorithm for further refined screening.

### 2.4. Molecular Dynamics Simulations of Docked Complexes

The MD were simulated to study the dynamical behavior of the top-scoring docked complexes using the GROMACS package [47]. Initially, amber force fields were applied using the Amber tool package [48]. GROMACS topology files were created by converting amber topology files using the AnteChamber Python Parser interface script. To get electrically neutral complexes, the complexes were solvated in a cubic box of water molecules, and appropriate counter-ions were added. The solvated system was minimized for about 10,000 steps using the steepest descent and conjugate gradient methods until the force on each atom was less than 100 kJ/mol/nm. The geometrically minimized systems were then subjected to isothermal molecular dynamics simulations. 

## 3. Results and Discussion

### 3.1. Outcomes of High Throughput Virtual Screening and Docking Studies

Human ApoE4, one of the most promising drug targets for treating AD, was virtually screened against approximately 0.2 million compounds of the ZINC database. The screened compounds were ranked according to their binding affinity, calculated as the scoring function called the Glide GScore. Of all compounds, a total of 10,000 compounds were identified from HTVS out of which those with a Glide score of less than −6.0 (64 compounds) were subjected to the Glide XP docking protocol. The top two scoring compounds and their properties are listed in Table 1. The values of the other docking parameters used for evaluating the selection criteria of the top-scoring ligands are shown in Table 2.

The top-scoring compound (4-imidazoleacetic acid hydrochloride; ZINC19735138; IAH) had a Glide score of −6.79 kcal/mol, while the second compound (2-methyl, 3-hydroxy-4,5-dihydroxymethylpyridin or pyridoxine hydrochloride; ZINC00049154; PHC) had a score of −6.76 kcal/mol. The results revealed that IAH had a stronger binding affinity for human ApoE4 protein than PHC. Both ligands interacted with the two catalytic triad residues of ApoE4 in addition to other neighboring residues of the active site. 

### 3.2. Binding Mode Analysis of Ligand-Docked ApoE4 Complexes

#### 3.2.1. ApoE4-IAH Complex

**Figure 1:**
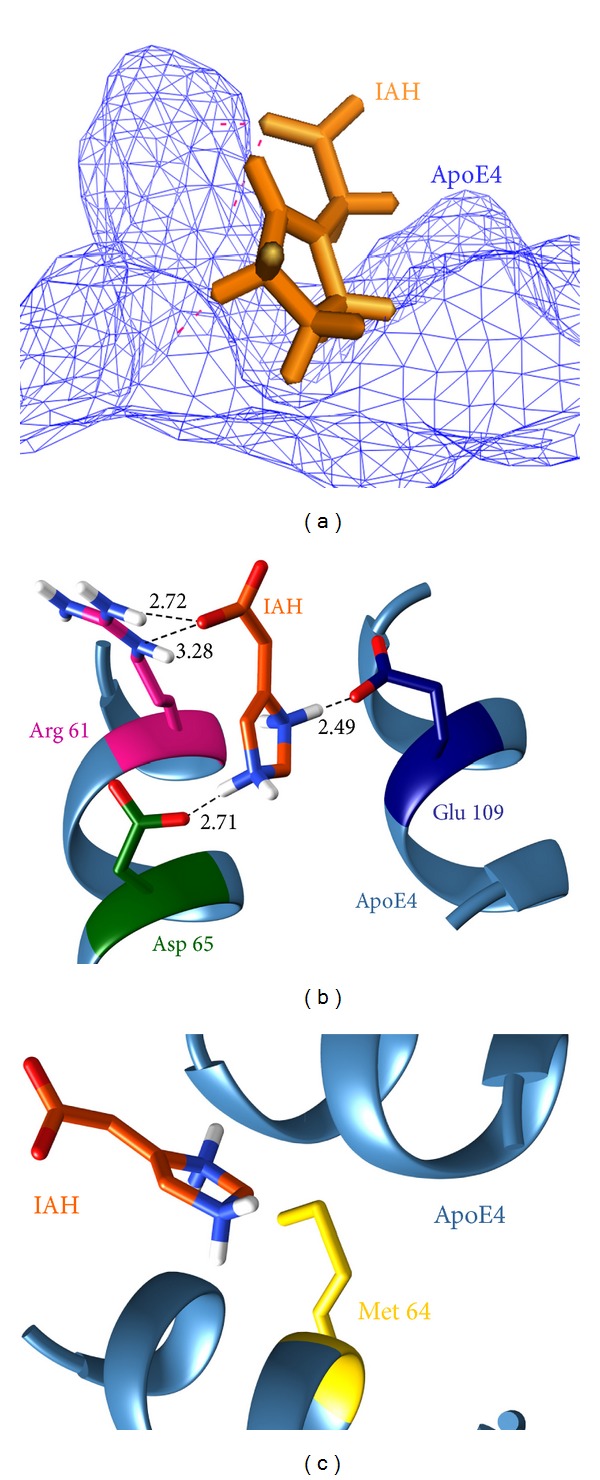
Molecular interactions between IAH (orange) and ApoE4 before MD simulations. (a) Position of IAH in the ligand-bound ApoE4 complex. (b) Hydrogen bond interactions. (c) Hydrophobic interactions.

In the case of the ApoE4-IAH complex, IAH interacted with the active site residues of ApoE4 (Figure 1(a)) with the formation of 3 hydrogen bonds and numerous hydrophobic contacts. Arg 61, Asp 65, and Glu 109 were the residues participating in hydrogen bond formation (Figure 1(b)). The NE and NH_2_ atoms of basic catalytic amino acid Arg-61 formed 2 hydrogen bonds (3.28 Å, 2.73 Å) with the O atom of IAH. Other hydrogen bonds (2.49 Å, 2.71 Å) were formed by atom N_1_ of IAH with the OE_2_ atom of acidic active site residue Glu 109 and OD1 of neighboring acidic residue Asp-65 and atom N2 of IAH. In addition, Met-64 was involved in hydrophobic interaction in the ApoE4-IAH complex (Figure 1(c)). Among all these interacting residues, Arg 61 and Glu 109 (part of the catalytic triad) are crucial amino acids and play a prominent role in abolishing the structural orientation of ApoE4. IAH bound to these residues, thus preventing interaction among them and improving the functionality of ApoE4. Various chemical properties of IAH were considered that supported its drug-likeness for AD treatment (Table 1). The topological polar surface area was reasonably high, which indicated that it can readily be absorbed in the human intestine and can penetrate the blood-brain barrier (BBB). In IAH, the presence of 12 heavy atoms and a high potential energy of 50.33 kcal/mol suggested that this ligand molecule has a good binding affinity for human ApoE4.

#### 3.2.2. ApoE4-PHC Complex

**Figure 2:**
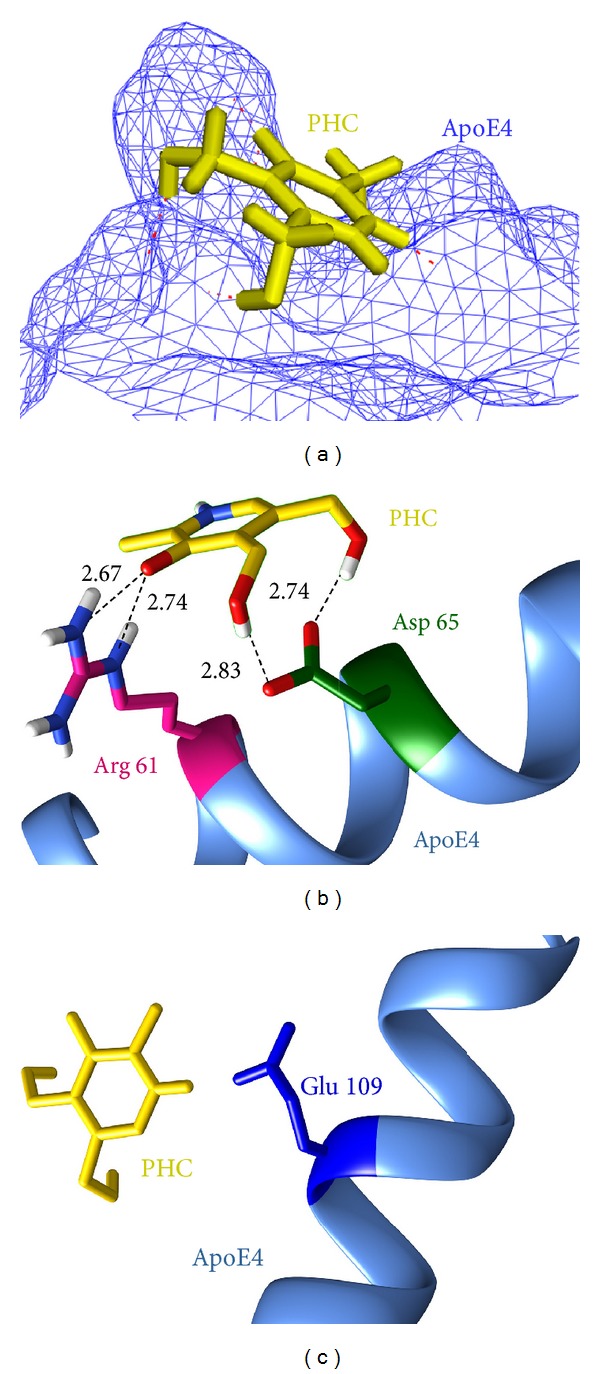
Molecular interactions between PHC (yellow) and ApoE4 before MD simulations. (a) Position of PHC in the ligand-bound docked complex. (b) Hydrogen bond interactions. (c) Hydrophobic interactions.

PHC is a single ringed structure with a molecular weight of 169.18 g/mol and lipophilicity value (log⁡*P*) of −0.55 at pH 7. The topological polar surface area of PHC was also considered as it is very useful for identifying drug transport properties, human intestinal absorption, and BBB infiltration. The presence of a reasonable number of heavy atoms (9) and a good potential energy of 74 kcal/mol suggest that PHC is capable of binding strongly with ApoE4 (Figure 2(a)). In this study, PHC formed 4 hydrogen bonds and 1 hydrophobic contact with human ApoE4. As can be seen in Figure 2(b), 2 hydrogen bonds were formed between the NH_2_ and NE atoms of active site residue Arg 61 and the O_3_ atom of PHC with bond length 2.67 Å and 2.74 Å, respectively, while 2 others were formed with the OD1 and OD2 atoms of the neighboring residue Asp 65 and O1 and O2 atoms of PHC with bond length of 2.83 Å and 2.74 Å, respectively. However, acidic amino acid Glu 109 of the catalytic triad was involved in making hydrophobic contact with PHC as illustrated in Figure 2(c). Of all these residues, Arg 61 and Glu 109 as part of the catalytic triad are responsible for the structural aberration in the human ApoE4 protein. These interactions of PHC with the crucial residues of ApoE4 suggest that this is a promising ligand that could correct the functionality of abnormal ApoE4.

### 3.3. Molecular Dynamics Simulations of Ligand-Bound ApoE4 Complexes

#### 3.3.1. Interaction Analysis of the ApoE4-PHC Complex

**Figure 3:**
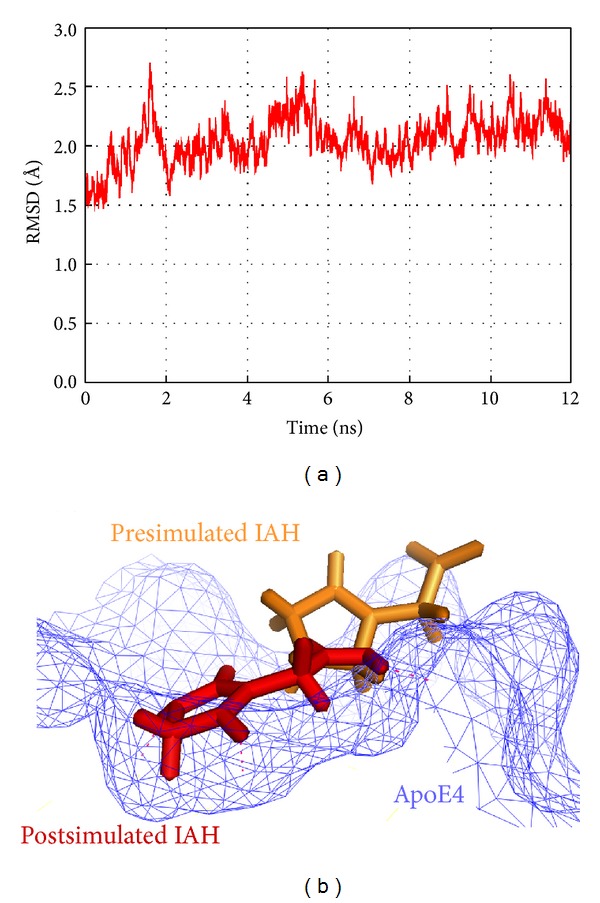
MD simulations trajectories: (a) RMSD trajectory of IAH in complex with ApoE4 obtained after MD simulations, (b) superimposition of pre-MD (orange) and post-MD (red) complexes of IAH with ApoE4.

**Figure 4:**
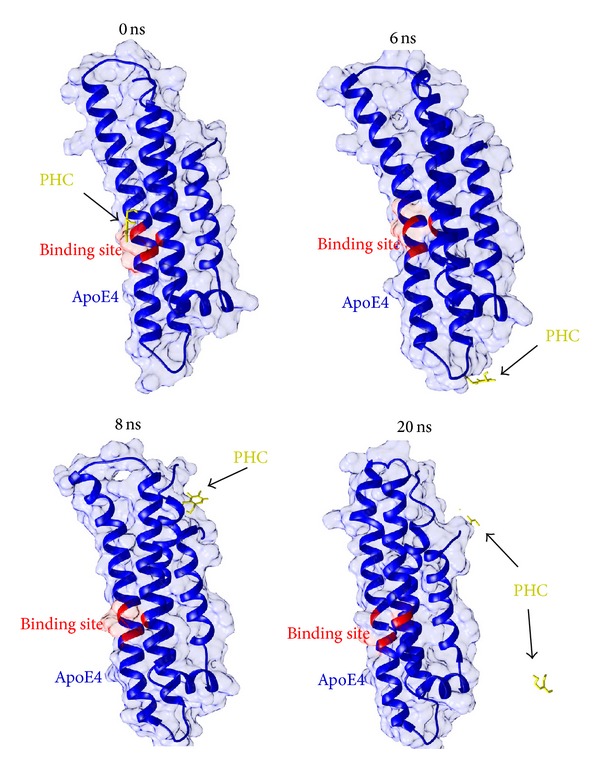
Snapshots depicting the binding instability of PHC with APoE4 during the MD simulations trajectory.

For further refinement and stabilization of both docked complexes, the MD were simulated using the GROMACS package. The simulation lengths used in the study were long enough to allow rearrangement of the side chains of the native and the ligand-complexed protein thus facilitating the most stable binding mode. As is evident in Figure 3(a), the backbone of the protein acquired stability after 8 ns with a root mean square deviation (RMSD) of only about 2.5 Å from its initial position. However, the MD simulations for ApoE4-PHC complex conducted for up to 24 ns revealed interesting results. PHC moved away from the binding site of ApoE4 during the simulations and lost all interactions formed in the initial docked pose.  Figure 4 illustrates the binding instability snapshots of PHC with ApoE4 during the simulation trajectory. During the MD simulations, the position of PHC in the ligand-bound complex was constantly altered. As can be seen from the snapshots at 6 ns and 8 ns, PHC moved far away from the binding site while staying at the surface of the protein. However, at 20 ns PHC was highly destabilized and split. Thus, it can be inferred that during the docking procedure the interactions of PHC with residues Arg 61, Arg 65, and Glu 109 of ApoE4 were only the result of static contacts. These pseudointeractions readily vanished when dynamics was considered in the study.

#### 3.3.2. Interaction Analysis of MD-Stabilized ApoE4-IAH Complex

**Figure 5:**
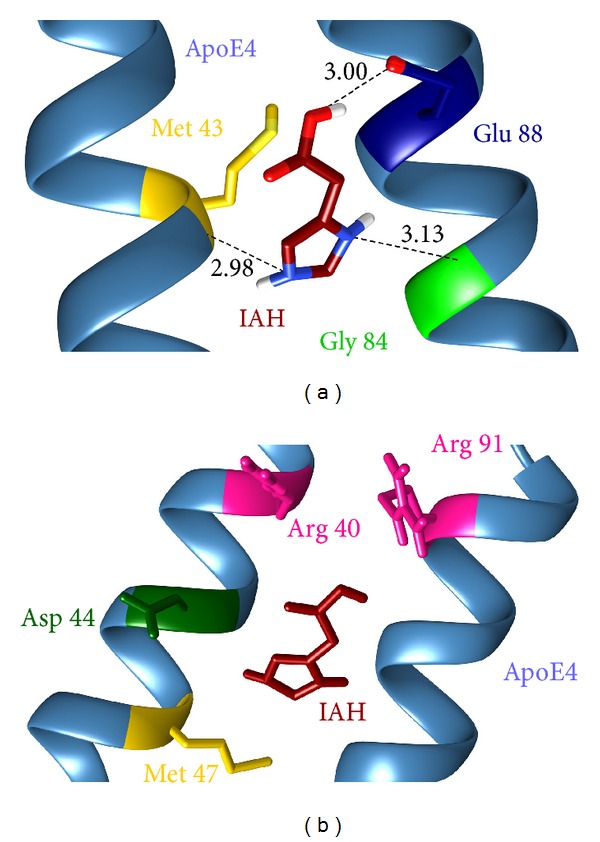
Molecular interactions between IAH (orange) and ApoE4 after MD simulations: (a) Hydrogen bond interactions and (b) hydrophobic interactions.

In the energetically stable ApoE4-IAH complex, the IAH molecule interacted with the residues Arg 61, Glu 109, and Arg 112 of the catalytic triad of ApoE4. The IAH molecule also formed contact with the residues Met 64, Asp 65, Met 68, and Gly 105. Though some deviation of IAH was observed from its initial position leading to a change in its binding mode, the binding was stable inside the ApoE4 cavity. A comparative analysis of the interaction profiles of ApoE4-IAH complex before and after the MD simulations is described in Table 3. The superimposition of the ligand IAH in the pre- and post-MD simulated complex structures inside the active site of ApoE4 is depicted in Figure 3(b). Initially, IAH formed 4 hydrogen bonds with the residues Arg 61, Asp 65, and Glu 109 of ApoE. After the simulations, 3 hydrogen bonds with the residues Arg 61 and Asp 65 had been replaced with 2 new hydrogen bonds involved with amino acids Gly 105 and Met 64. The hydrogen bond with the residue Glu 109 remained consistent with a slight change in the bond length (Figure 5(a)). The only hydrophobic contact with Met 64 was present in IAH-bound ApoE4 before MD disappeared during the MD simulations. However, after the MD simulations IAH formed strong hydrophobic contacts with 4 residues of ApoE4 (Figure 5(b)). The stability of IAH in the binding pocket of APoE4 is prominently governed by these hydrophobic contacts. After the MD simulations, IAH acquired a more stable conformation within the active site of ApoE4 by placing itself deep inside the cavity. 

## 4. Conclusion

In the present work, we screened two top-scoring compounds, IAH and PHC, which possess high Glide XP scores of −6.79 kcal/mol and −6.76 kcal/mol, respectively, against human ApoE4. These compounds interacted with the catalytic triad residues of ApoE4 that are crucial for maintaining its aberrant structure. The binding of these ligands suggests that they have a strong mechanistic ability to correct the pathological structural orientation of ApoE4 by preventing repulsion between Arg 61 and Arg 112, thus inhibiting the formation of a salt bridge between Arg 61 and Glu 255. The chemical properties of these potent structure-correctors are in line with the stipulated requirements of drug-like compounds for further experimental analysis. After the MD simulations, the interactions formed by IAH were consistent. However, a comparison between the conformations obtained from docking and that from molecular dynamics simulations for the second ligand PHC revealed substantial changes in binding conformations. Our simulation results indicate that the initial receptor-ligand interaction observed after docking can be limited due to the receptor rigid docking algorithm and that the conformations and interactions observed after the simulation runs are more energetically favored and should be better representations of the derivative poses in the receptor. Our detailed binding analysis of IAH substantiated by its dynamic structural stability provides considerable evidence for use as a potent natural lead against Alzheimer's. Results from this study would also be helpful in designing novel neuroregenerative drugs with improved binding properties and low toxicity.

## Figures and Tables

**Table 1:** Physical properties of potential structure correctors identified using virtual screening.

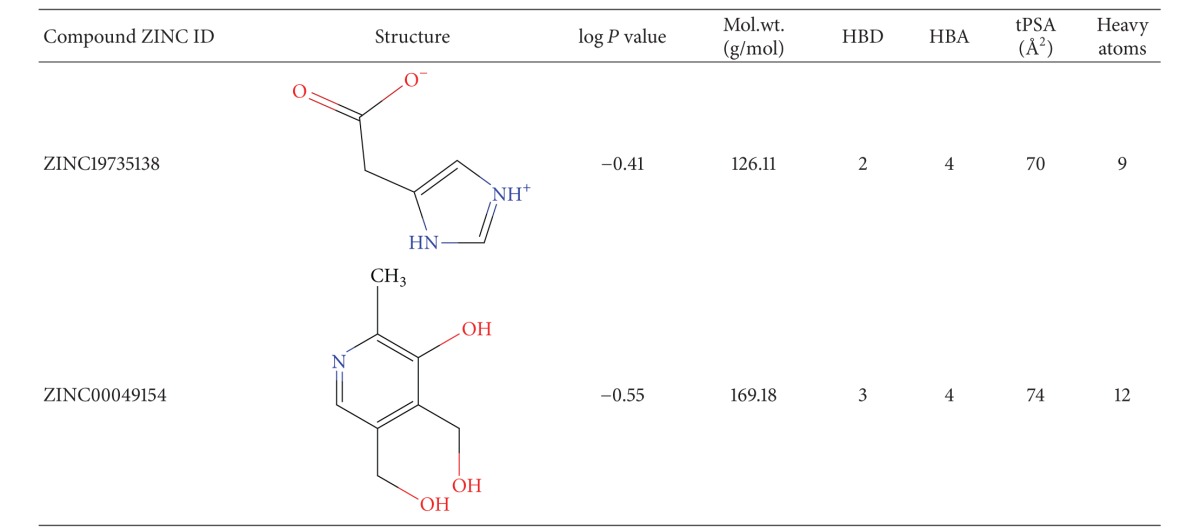

Mol.wt.: Molecular weight, HBD: hydrogen bond donor, HBA: hydrogen bond Acceptor, tPSA: topological polar surface area.

**Table 2:** Binding affinity scores and energies of ApoE4 in complex with IAH and PHC.

Compound	ZINC ID	Docking score	XP Gscore	Glide ligand efficiency	Glide evdw	Glide emodel	Glide energy
IAH	ZINC19735138	−6.79	−6.79	0.75	−3.28	−23.90	−28.96
PHC	ZINC00049154	−6.76	−6.76	−0.56	−6.18	−32.97	−26.97

**Table 3:** Molecular interactions present in pre- and post-MD simulated IAH-bound ApoE4 complexes.

ApoE4-IAH complex	Residues participating in hydrogen bonding	Residues governing hydrophobic contacts	Hydrogen bond length (Å)
Pre-MD	Arg-61 Asp-65 Glu-109	Met-64	3.28, 2.73 2.71 2.49
Post-MD	Met-64 Gly-105 Glu-109	Arg-61, Asp-65, Met-68, Arg-112	2.98 3.14 3.08
